# Trade‐Offs of Conservation Fencing in Western Serengeti: Enhancing Agricultural Security While Navigating Unintended Consequences on Land‐Use Dynamics

**DOI:** 10.1002/ece3.73674

**Published:** 2026-05-17

**Authors:** Michael Honorati Kimaro, Milenka Ishasha Sloots, Kristen Denninger Snyder, Noel Latiaeli Mbise, Victor Alexander Kakengi, Walter Di Nicola, Han Olff

**Affiliations:** ^1^ Groningen Institute for Evolutionary Life Sciences University of Groningen Groningen the Netherlands; ^2^ Tanzania Research and Conservation Organization Morogoro Tanzania; ^3^ Grumeti Fund Mugumu Mara Region Tanzania; ^4^ Department of Fish, Wildlife & Conservation Biology Colorado State University Fort Collins Colorado USA; ^5^ Tanzania Wildlife Research Institute Arusha Tanzania; ^6^ Department of Wildlife Ecology and Conservation Biology Albert Ludwig University Freiburg Freiburg Germany; ^7^ Department of National Park Monitoring and Animal Management Bavarian Forest National Park Grafenau Germany

**Keywords:** community, electric‐conservation‐fencing, land‐cover, land‐use, livelihoods

## Abstract

Protected areas (PA) in African savannas support biodiversity and ecosystem services, but are increasingly surrounded by hard land‐use transitions as surrounding human populations grow. Fencing PAs is a potential, yet contested, response to mitigate growing human–wildlife conflicts at their edges. We test the consequences of such conservation fencing for communities neighboring PAs in the Serengeti ecosystem, Tanzania, in a multi‐year before‐and‐after study of an adjacent fenced (Ikorongo Game Reserve) and unfenced (Serengeti National Park) area, using remote sensing, ground transects, and community interviews. We found that conservation fencing contributed to changes in cropland or livestock grazing coverage. In addition, conservation fencing did not have a clear impact on the spatial zonation of specific crop types. Positive perceptions expressed by community members towards the conservation fence were: (i) more success with crop farming due to less time needed for guarding crops against nocturnal elephant damage, (ii) youth shifting their focus from (risky) poaching to crop farming and small business. Negative perceptions included: (i) more land use conflict because grazing lands close to the fence became cropland, where few livestock keeping households with large herds moved to the unfenced site, (ii) restricted access to resources (livestock grazing, thatch grass, water) within the reserve, despite the illegality of their use. We conclude that conservation fencing can strongly benefit farmers near PAs while protecting wildlife, particularly along hard boundaries, but also risks leakage effects (unwanted spillover into nearby areas). Implementing conservation fencing, therefore, requires careful consideration of the larger socio‐ecological landscape, for instance, by accounting for current land uses and changes in the spatial availability of resources resulting from fencing.

## Graphical abstract

Fencing enhanced security in cropland livestock grazing land. Fence did not have a significant change in crop zonation patterns. While local communities are largely happy with the fence, fencing triggered land‐use conflicts and the resettlement of a few livestock keepers with large herds of livestock to nearby unfenced sites.

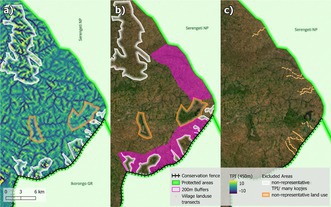

## 1 Introduction

Across the African savannah biome, rural human population growth is driving increasing pressure on land near protected areas (Jones et al. 2018). As more people expand their farms and move livestock closer to these protected areas (PAs), the once‐gradual or “soft” transitions between human‐dominated landscapes and wildlife habitats are being replaced by abrupt, “hard” boundaries marked by sudden shifts in land cover. At these hard boundaries, intensive land uses (such as crop cultivation and high livestock densities) often extend right up to PA borders, increasing competition for space and resources, usually leading to heightened conflict between humans and wildlife (Adams and Hutton 2007; Ogutu et al. 2011; Veldhuis et al. 2019). This conflict can exist at multiple levels, from disputes over direct tangible impacts (like crop loss or damage by wildlife, or livestock grazing within protected areas) to underlying conflicts fuelled by past grievances and mistrust, and deep‐rooted conflicts where wildlife issues become tied to identities, values, and broader social tensions (Denninger Snyder et al. 2023; IUCN SSC 2023). In response to rising tensions and increasing conflicts, there is an urgent need for strategies and management interventions that balance human livelihoods with biodiversity conservation at the edges of PAs.

Conservation fencing (separating communities and neighboring communities by a fence) has been increasingly adopted as a conservation tool, especially in regions with high human population densities and agricultural lifestyles close to protected areas (Bariyanga et al. 2016; Bissett et al. 2012; Graham, Douglas‐Hamilton, et al. 2009; Thouless and Sakwa 1995). We define conservation fencing as the physical restriction of movement across a protected area boundary. Unlike enclosure fences around private pastures or infrastructure fences, which largely fragment landscapes and negatively affect both wildlife and open‐range pastoralists (Boone and Hobbs 2004; Gadd 2012; Løvschal et al. 2017), conservation fencing is generally intended to produce positive outcomes for both people and wildlife. These positive outcomes include preventing poaching and illegal incursions into the PA, thereby protecting the animal and plant species within (Hayward and Kerley 2009). Conservation fences have also been shown to prevent wild animals from damaging crops, depredating livestock, or harming people; thus, they also protect large herbivores and carnivores from retaliatory attacks (Davies et al. 2011; Kioko et al. 2008; Sapkota et al. 2014).

Despite the potential benefits of conservation fencing, there is debate on balancing its socio‐economic and ecological costs and benefits. Ecologically, if not well‐positioned, conservation fencing may disrupt ungulate seasonal migrations, cause large‐scale direct wildlife deaths (Gadd 2012), increase predation risk, reduce genetic diversity, and necessitate ongoing management efforts (Hayward and Kerley 2009; Somers and Hayward 2012). Socio‐economically, fences are expensive to construct and exclude local communities from accessing PAs and the resources within them that they have historically had access to (Brockington et al. 2006; Hoole and Berkes 2010; Lindsey et al. 2012). While the ecological and social impacts of conservation fencing are relatively well studied, far less is known about how land use evolves after a fence is installed. Theoretically, by reducing crop damage and lowering farmers' personal risk, conservation fences can encourage farmers to cultivate land closer to the boundaries of protected areas (Bariyanga et al. 2016). Empirical evidence supports this: Cilliers et al. (2025) found higher agricultural cover immediately outside fenced protected areas compared to unfenced ones. But the spill‐over effects if only part of a PA is fenced have, to our knowledge, not been studied. Indeed, although fences are intended to reduce conflict, they may unintentionally exacerbate human–wildlife conflict by displacing wildlife movements into nearby unfenced areas and altering land‐use patterns and social dynamics, thereby heightening underlying tensions in communities. Therefore, understanding the landscape‐wide consequences of conservation fencing is key to its potential success.

**FIGURE 1:**
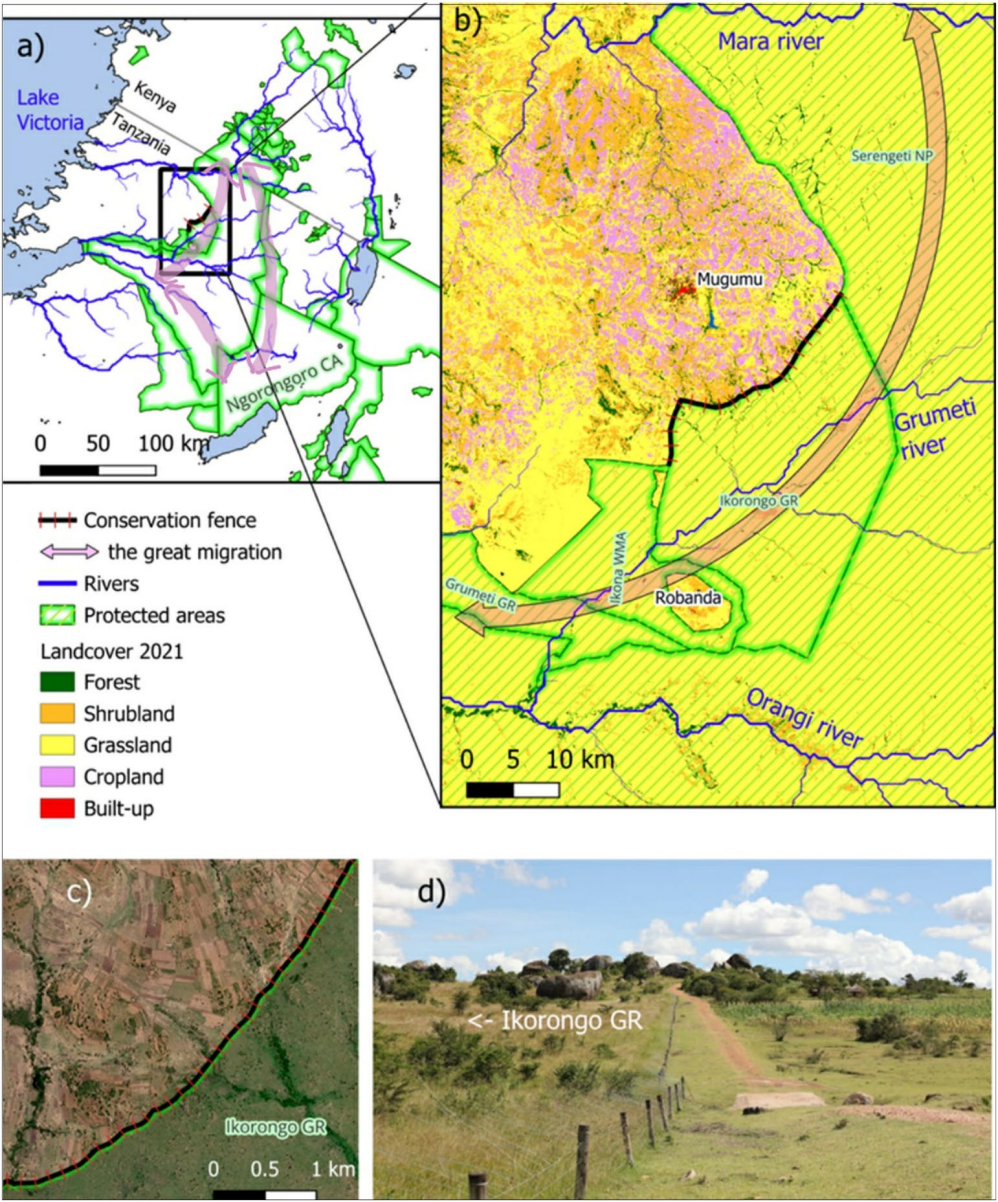
The figure shows the electric conservation fence built along the boundary of Ikorongo Game Reserve in northern Tanzania, the neighboring protected areas of the GSME (a), major rivers, and the ‘great migration’ route of wildebeest and zebra from the Serengeti Plains to the Mara River (a, b). A detailed landcover image (b), as shown by the 2021 land cover (ESA WorldCover v200) and satellite image (c) (from October 2023, Maxar, Planet.com), highlights the hard boundary of IGR, separated from adjacent cropland by the conservation fence. Additionally, the photograph (d) captures the electric conservation fence near crop fields and houses (April 2023, photo by Han Olff).

Despite ongoing debate over fencing issues, conservation fencing is increasingly adopted in Sub‐Saharan Africa (Bissett et al. 2012; Thouless and Sakwa 1995). East African nations, particularly Tanzania, have been cautious about adopting conservation fencing due to unresolved questions about its efficacy and potential drawbacks. This hesitation stems mainly from the question of when to install it, particularly in areas where wildlife relies on village lands for seasonal movements between protected areas, which serve as critical migratory routes for refuge and breeding, making fencing potentially disruptive to ecological connectivity. However, recent developments in Tanzania have shifted this trend, particularly with the introduction of the National Human‐Wildlife Conflict Mitigation Strategy, which highlights the need for research into electric fences as a potential tool for mitigating human–wildlife conflict (URT 2020). In 2019, the Ikorongo Game Reserve (IGR) management in the western Serengeti reintroduced a group of critically endangered Eastern black rhinoceros ( *Diceros bicornis* ) to restore biodiversity and boost tourism. To protect these rhinos, the IGR management, in collaboration with the Grumeti Fund of Tanzania, constructed a 30‐km electric conservation fence along the northern reserve's boundary. Although the fence was primarily intended to keep the rhinos within the IGR, it was also designed to reduce crop damage caused by elephants in nearby village lands (Grumeti Fund 2020, 2025). This area lies within the optimal conditions for considering fencing: the village lands bordering the IGR are heavily cultivated and densely populated, and the major wildlife migrations—primarily wildebeest and zebra—remain entirely within the protected area and do not cross its boundary (Figure 1a). Completed in April 2020, this fence provides a unique opportunity to study the impacts of conservation fencing on LULC changes in villages bordering the Ikorongo Game Reserve, as well as analyzing the adjacent unfenced sites bordering Serengeti National Park.

In this study, we investigate the impact of this conservation fence system on village land‐cover types across four key categories: croplands, livestock grazing areas, thicket vegetation, and woodland/forest. This analysis compares the pre‐fence and post‐fence periods, as well as currently fenced sites (villages bordering Ikorongo Game Reserve) with spatially adjacent unfenced sites (villages bordering Serengeti National Park). Additionally, this study examines spatial shifts in the coverage of specific crops commonly cultivated in the study area and local communities' perceptions of potential land‐use and land‐cover changes.

## 2 Materials and Methods

### 2.1 Study Area

This study was conducted along the northern border of the Ikorongo Game Reserve (IGR) and the adjacent western border of the Serengeti National Park (SNP), located in the northwestern part of the Greater Serengeti‐Mara Ecosystem (GSME), in northern Tanzania (Figure 1). The GSME is a transboundary ecosystem spanning Tanzania and Kenya, characterized by a semi‐arid climate, with seasonal rainfall patterns that influence vegetation growth and wildlife movements (Hopcraft et al. 2015). The GSME is particularly renowned for the largest terrestrial mammal migration on Earth, the annual movement of approximately 1.3 million wildebeests ( *Connochaetes taurinus* ), alongside a rich diversity of other wildlife, including zebra ( *Equus quagga* ). The mass migration of wildebeests moves from the Serengeti Plains in the south, where they have their wet‐season calving areas, to the vicinity of the Mara River in the north, where they find their dry‐season refuge. The area also harbors the endangered elephants ( *Loxodonta africana* ) and the critically endangered black rhinoceros (Sinclair et al. 2000; Ogutu et al. 2011).

The IGR is a critical component of this migratory ecosystem, serving as a key movement corridor for wildebeest and zebra migrations (Figure 1). The IGR covers approximately 600 km^2^. The SNP covers 14,763 km^2^ and is characterized by a mosaic of habitats, including grasslands, woodlands, and rocky outcrops, which provide essential resources for wildlife (Nyahongo et al. 2009; Msoffe et al. 2011). The village lands adjacent to the protected area boundaries in the study area—including those along the northern boundary of IGR and the boundary of SNP—are inhabited by local communities who depend on subsistence farming and livestock grazing (Kavwele et al. 2022), activities that extend up to the boundaries of these PAs (Estes 2012; Figure 1), and have moved closer to these boundaries over time (Veldhuis et al. 2019), including illegal entry with livestock in the PAs for grazing (Denninger Snyder et al. 2019; Veldhuis et al. 2019). This proximity has led to frequent human–wildlife conflicts, particularly involving elephants that raid crops and threaten local livelihoods (Denninger Snyder et al. 2023).

The fence along the IGR boundary was completed in April 2020. It was designed not only to protect rhinos but also to deter elephants from entering village lands, thereby reducing crop damage and conflicts (Grumeti Fund 2020, 2025). We compare fenced sites (villages bordering IGR) and unfenced sites (villages bordering Serengeti National Park, SNP), allowing a comparative analysis of land use and land cover (LULC) changes during the pre‐fence and post‐fence stages, as well as between current fenced and unfenced sites.

### 2.2 Data Collection

To assess the fence's impacts on Land Use and Land Cover (LULC), we employed three complementary methods: LULC mapping and change analysis using remote sensing, field transects on village land, and community interviews.

#### 2.2.1 LULC Classification Using Remote Sensing

To identify LULC trends, we conducted a LULC classification for the 2 years preceding the construction of the electric fence along the IGR boundary (2018 and 2019) and the 2 years following (2021 and 2022) using Google Earth Engine. Sentinel‐2 imagery for the study area is only available from 2018 onward, so selecting these years ensured a balanced temporal window on both sides of the fence construction. The classification was conducted using Google Earth Engine with satellite data from Sentinel‐2 and Sentinel‐1. Both Sentinel 1 and Sentinel 2 data were used, as combining radar imagery (which captures vegetation structure) with optical imagery (which captures vegetation and soil color) is more effective in LULC classification than using optical imagery alone (Lopes et al. 2020). We used the VV and VH bands for Sentinel‐1 in ascending orbit. For Sentinel 2, the visible light and infrared‐type bands were used (B1‐B8A, B11, B12). In the year 2018, no Sentinel 2 2A scenery was available, so the Sentinel 2 1C product was converted into surface reflectance using the SIAC atmospheric correction application for Google Earth Engine (Yin et al. 2022). For each year, Sentinel‐1 and Sentinel‐2 data were filtered into three time periods, with a cloud cover threshold of < 30%. The periods were selected to capture key seasonal stages that strongly influence spectral signatures: the period in which crop fields are usually plowed (February), a wet season image when vegetation is most green and vigorous (May), and a dry season image when vegetation is at its driest and most sparse (August) (see Appendix A). Cloudy pixels were masked out from each of these image collections. Combining these three periods into a single composite image improves class separability, as different classes exhibit distinct spectral signatures at various times of year. By incorporating imagery from several key periods, it becomes easier to distinguish classes that may appear similar in a single season.

Furthermore, vegetation indices (i.e., NDVI, NDBI, BSI, and MNDWI) were calculated to improve vegetation type classification (Gandhi 2021; Chaves et al. 2020). The image collections for the 3 periods of each year were then composited into a single image using the median reducer, resulting in 4 final images, one for each analyzed year. To obtain more precise results from these four images, speckle in the 10‐m‐resolution Sentinel‐1 images was reduced by smoothing within a 20 m circular radius around each pixel. Finally, the four images were concatenated into a single multi‐temporal composite scene, containing 48 bands from all four initial images.

To generate accurate LULC maps, training polygons were created using ground‐truth field measurements collected in the study area from April to May 2022. During this period, the coordinates of points within LULC types larger than 10 × 10 m were recorded, along with their corresponding classifications. The classification categories used were: Built‐up, Bare, Water, Short Vegetation, Woodland, Thicket, Kopje, and Cropland. Cropland included all crop types and plowed fields. Based on these coordinates, familiarity with the study area, and true‐color and false‐color satellite images, about 350–400 training polygons were drawn in QGIS v3.28 (QGIS Development Team 2023) for each analyzed year.

**FIGURE 2:**
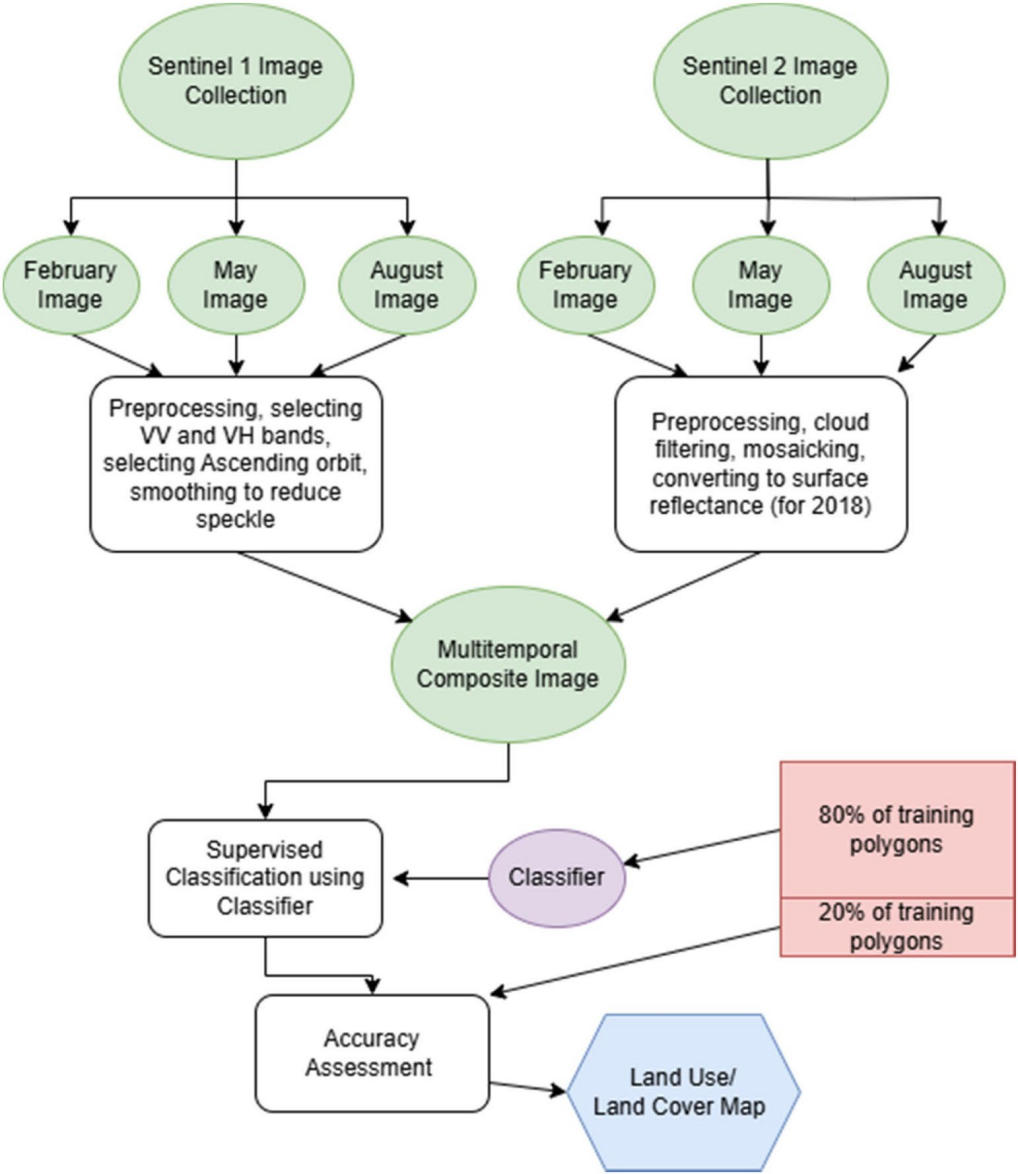
Flowchart of the process used to create an annual LULC map for the study area.

In Google Earth Engine, we trained a Random Forest classifier for each year using 80% of that year's training polygons. This classifier was then used to classify the multi‐temporal composite image created from Sentinel‐1 and Sentinel‐2 data at a 20 m resolution (see Appendix B). The remaining 20% of the training polygons were used to develop a classification error matrix, from which the kappa accuracy was calculated. The process described above is summarized in Figure 2.

**FIGURE 3:**
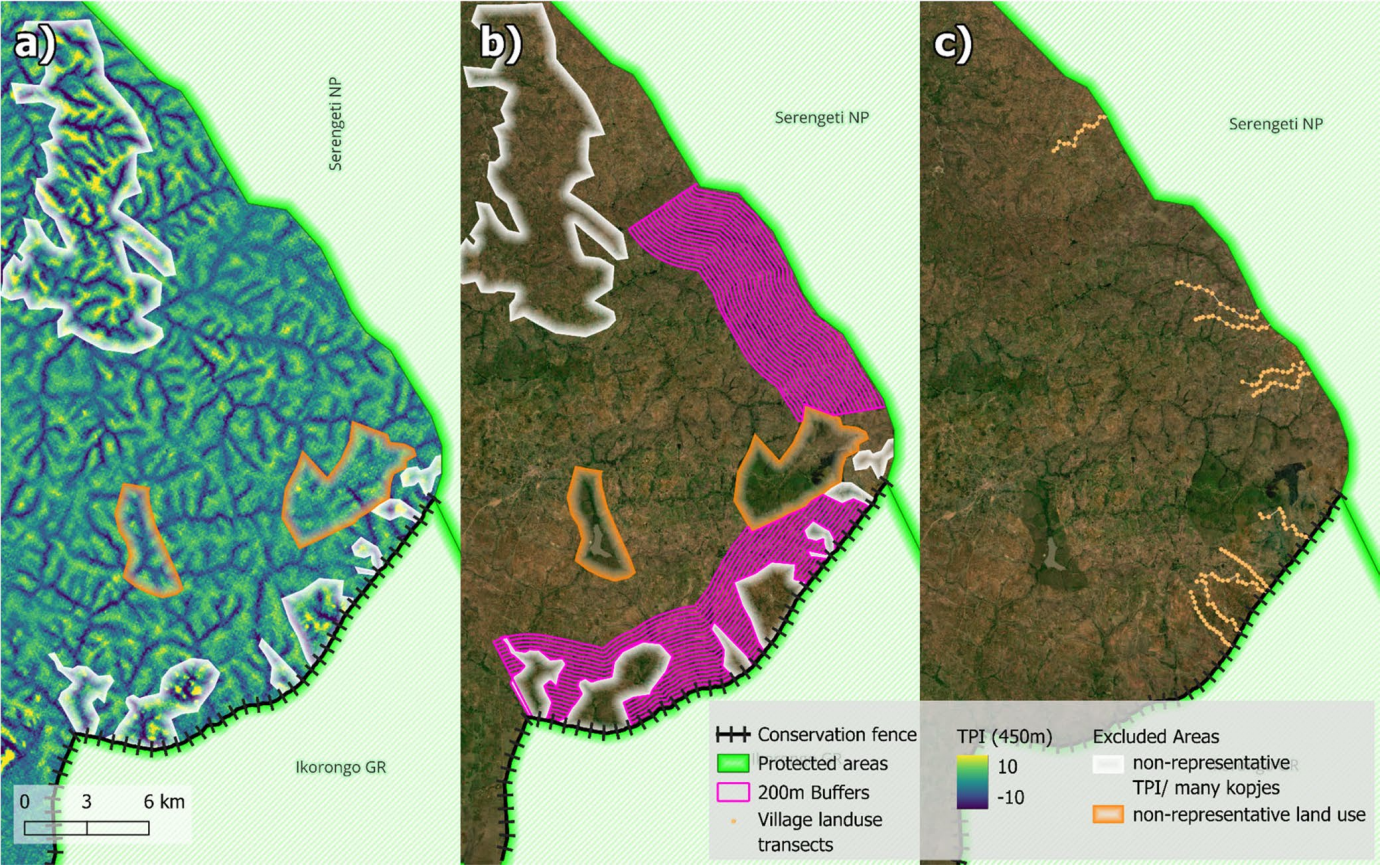
200 m buffers with areas excluded due to diverging topographic position index (deep valleys and rocky “kopjes” areas (a), due to nonrepresentative land use (i.e., a water reservoir and Tabora B Prison) (a), and (b), while c) shows the location of village land transects used for sampling land use in the field.

Finally, using the classified LULC maps for each year, we extracted the number of 20 × 20 m pixels for two buffer sets: one bordering the fenced IGR and the other bordering the unfenced SNP. These buffers were each 200 m wide, starting from the PAs' boundaries (0 m) and extending up to 4000 m, about 22 and 14 km along the IGR and SNP PA boundaries, respectively. Areas with nonrepresentative land use (i.e., prison grounds, high presence of kopjes) and diverging topographic position index were excluded (Figure 3a,b). For each buffer, we then calculated the proportion of area covered by the different analyzed land cover types based on the number of pixels investigated.

#### 2.2.2 Village Transects

To allow for more detailed individual crop type analysis that cannot be determined from satellite imagery, in April/May 2022 and 2023, we set up 10 transects of 3 km each, 5 in village lands bordering fenced (IGR) and 5 in unfenced (SNP) sites (Figure 3c). A total of 15 sampling sites of 20 m radius were placed at intervals of 200 m starting from the protected area boundary, yielding 150 plots of 1256 m^2^. The sampling sites were placed on a top‐slope or middle‐slope catena position. We also excluded the Tabora B prison grounds from the transects, removing two sampling points from one transect, leaving us with 148 sites. At each site, we visually estimated the percentage cover of each land cover type within a 20 m radius plot.

#### 2.2.3 Focus Group Discussions and Key Informant Interviews

Local leaders from four of the five villages (i.e., Miseke, Kazi, Rwamchanga, and Bonchugu), located along the fence line, were selected to share their perspectives on how the fence has influenced land‐use and land‐cover changes and the communities' livelihoods. Leaders from Park Nyigoti village, which also borders the fence, did not attend the focus group discussion despite being invited. All FGD participants were agropastoralists who keep livestock and cultivate crops. Two focus group discussions (FGDs) were conducted in December 2024. The FGDs were facilitated by one of the coauthors, who also serves as the Principal Investigator of this fencing project at the Tanzania Wildlife Research Institute (TAWIRI). The facilitator is based at TAWIRI's headquarters in Arusha, Tanzania. Each FGD consisted of six participants, comprising three village leaders, two women, and one young man (a youth). These women and youths were encountered at random while visiting the villages and invited to participate because all the village chairs were older men. Therefore, the two FGDs included a total of 12 participants. Key informants, including one politician from the ruling party and two district‐level government officials, were also selected to contribute their insights. Existing thematic categories involved in the discussion were (i) briefing participants on data collected and preliminary findings; (ii) ongoing research and monitoring efforts; (iii) recording testimonials on the impact of the fence on the socioeconomic aspects of different community groups (i.e., crop farmers, livestock keepers, women, youths); (iv) and recording testimonials on the impact of the fence on cultural practices (i.e., way of housing and cultural values). However, we agreed on major themes and related sub‐themes focusing on land‐use and land‐cover issues relevant to this study (Appendix D). Verbal consent was obtained from all participants before discussions or interviews to comply with the ethical guidelines required by the Commission of Science and Technology of Tanzania (COSTECH). All data were collected and stored securely in accordance with the University of Groningen's regulations and guidelines, the Netherlands. To ensure the anonymity of interviewees, no names or contact details were documented or recorded, and no identifying information was stored.

### 2.3 Data Analysis

#### 2.3.1 LULC Classification Using Remote Sensing

Using the LULC classification maps, we assessed how the proportion of different land cover categories varied spatially and temporally using R 4.4.1 (R Core Team 2024) and RStudio (Posit team 2025). To do this, we calculated the difference in land cover proportion (on a pixel basis) between unfenced SNP and fenced IGR (SNP‐IGR) for each 200 m buffer at distances 0–4000 m from the PA boundary for each year. This resulted in 20 data points per year and per land‐cover variable, scaled to kilometers by dividing by 1000 using the “dplyr” package in R (Wickham et al. 2023). This variable highlights differences in land use between IGR and SNP rather than absolute proportions, thereby minimizing the impact of year‐specific classification inconsistencies. For each year and land cover type, we fitted a linear regression model to assess the effect of distance from the PA boundary on the proportion of that land cover type. To better identify changes in LULC close to the boundary, we also calculated the mean and standard error of area covered by each LULC type at distances 0–1.4 km from the PA boundary in the two PA village lands for the period before fencing (2018–2019) and after fencing (2021–2022) by multiplying the number of pixels of each LULC type by the size of the pixel (0.004 km^2^). We selected the 0–1.4 km range because, based on our regression model of differences in proportion, this interval captured the distance over which agricultural cover between fenced and unfenced boundaries diverged most strongly.

#### 2.3.2 Village Transects—Crop Types Variability

Because data from the village land transects were collected only after fencing (2022 and 2023), we analyzed how crop types varied spatially with increasing distance from the PA boundary, comparing village lands bordering the fenced IGR with those bordering the unfenced SNP. Based on the literature, the crops preferred by elephants are maize, millet, and sorghum (Compaore et al. 2020; Matsika et al. 2020; Montero‐Botey et al. 2021). We analyzed how these elephant‐preferred crops (including cassava and sweet potatoes) varied spatially across all LULC categories (listed in Appendix C). We aggregated percentage covers for each transect and crop type into three distances to boundary categories: (0–1, 1–2, 2–3 km). To assess differences in vegetation cover across distance categories and boundary types, we fitted a linear mixed‐effects model using the lme4 package in R. The model included fixed effects for distance to the protected area boundary categories, boundary status (fenced vs. unfenced), and transect as a random effect. We also tested whether including the interaction between distance to the boundary and boundary type improved the model by comparing AICs and testing the interaction's significance. We used the emmeans (Searle et al. 1980) package to perform Sidak post hoc comparisons of estimated marginal means for each combination of distance category and boundary status.

##### 2.3.2.1 Focus Group Discussions and Key Informant Interview Analysis

We employed qualitative content analysis by transcribing video recordings, extracting key points from focus group discussions and key informant interviews, and summarizing them in an Excel spreadsheet. The contents were translated from Swahili into English. In this study, we extracted information related to land‐use and land‐cover themes. This involved reorganizing the discussions and interviews into themes and sub‐themes, and extracting meaningful insights and illustrative quotes by manually watching and listening to the videos (Elo and Kyngäs 2008) to gain a deeper understanding of participants' perspectives and concerns. A single author conducted content analysis.

## 3 Results

### 3.1 LULC Classification Using Remote Sensing

**FIGURE 4:**
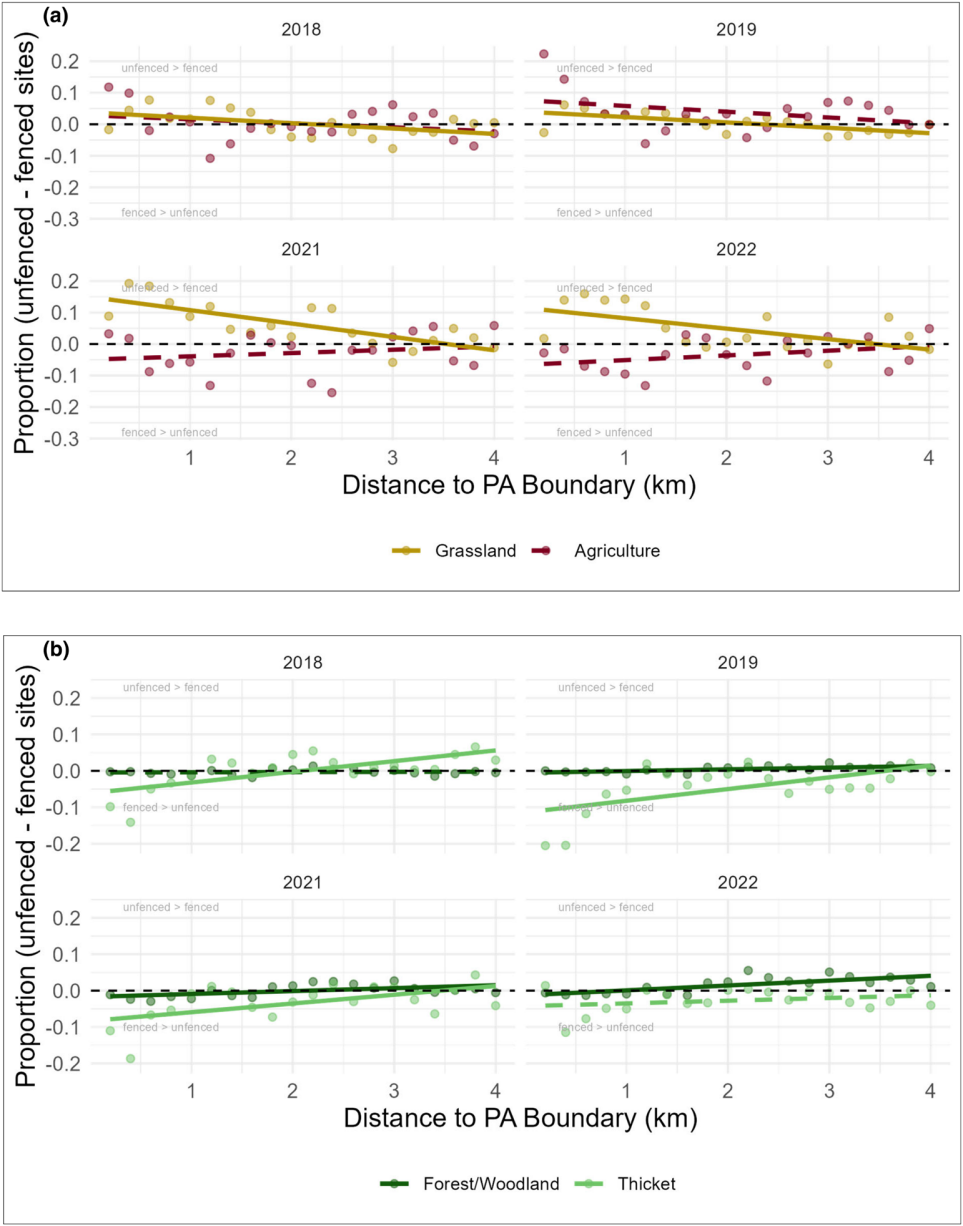
(a) Difference in proportions of agricultural land and grassland coverage between a fenced and an unfenced site during the pre‐fence stage (2018–2019) and post‐fence stage (2021–2022) relative to the distance (measured outside the PA) to the protected area boundary within the 4 km range inside the village lands. Negative proportions imply a higher abundance of that land cover type in the fenced site compared to the unfenced site. Positive proportions indicate the opposite. Solid and dotted lines represent significant or nonsignificant relationships between differences in proportion and distance to the boundary, respectively. (b) Difference in proportions of woodland and thicket coverage between fenced and unfenced sites during the pre‐fence stage (2018–2019) and post‐fence stage (2021–2022) relative to the distance to the protected area boundary within the 4 km range inside the village lands. Negative proportions imply a higher abundance of that land cover type in the fenced site compared to the unfenced site. Solid and dotted lines represent significant or nonsignificant relationships between differences in proportion and distance to the boundary, respectively.

During the pre‐fence stage (in 2018 and 2019), proportions of agricultural cover were generally similar across village land bordering SNP and IGR, with SNP‐IGR proportions close to zero. However, the proportion of agricultural cover close to the boundary was slightly higher in SNP village lands (Figure 4a). Agricultural area cover remained stable in IGR close to the boundary (0–1.4 km), going from 1.12 ± 0.034 km^2^ before fencing to 1.13 ± 0.023 km^2^ after fencing, but decreased after the fencing period in SNP (1.75 ± 0.037 km^2^ to 1.46 ± 0.024 km^2^). The proportion of grassland cover across all distances to the boundary was also relatively equal in IGR and SNP village lands (Figure 4a). However, in the years during the post‐fence stage (in 2021 and 2022), the proportion of cropland area was higher in fenced sites compared to unfenced sites. Furthermore, close to the protected area boundary (0–1.4 km), the proportion of grassland was higher in the unfenced sites than in fenced sites, while further from the boundary, grassland proportions in the two areas were similar (Table 1; Figure 4a). The cover of grassland remained stable after fencing in IGR (0.07 ± 0.004 km^2^ to 0.08 ± 0.010 km^2^), but increased after fencing in SNP (0.17 ± 0.014 km^2^ to 0.34 ± 0.026 km^2^). Across all years, *β*‐coefficients of agricultural cover are small (ranging between −0.018 and 0.015) and statistically nonsignificant (*p* > 0.05; Table 1). Grassland showed a stronger pattern, with significant *β*‐coefficients ranging from −0.017 to −0.042 (*p* < 0.05; Table 1).

**TABLE 1:** Coefficients from the linear models conducted from 2018 to 2022, showing the effect of distance from the PA boundary on the difference in land‐use coverage proportions between fenced and unfenced sites.

LULC type	Year	Estimate	Standard error	*t*	*p*
Agriculture	2018	−0.013	0.010	−1.206	0.243
	2019	−0.018	0.012	−1.155	0.139
	2021	0.010	0.012	0.845	0.409
	2022	0.015	0.010	1.511	0.148
Grassland	2018	−0.017	0.007	−2.369	**0.029**
	2019	−0.017	0.005	−3.625	**0.002**
	2021	−0.042	0.009	−4.818	**0.000**
	2022	−0.033	0.010	−3.179	**0.005**
Forest/woodland	2018	0.000	0.002	0.344	0.735
	2019	0.005	0.001	3.837	**0.001**
	2021	0.008	0.003	2.824	**0.011**
	2022	0.013	0.003	4.262	**0.000**
Thicket	2018	0.030	0.007	4.047	**0.001**
	2019	0.032	0.010	3.164	**0.005**
	2021	0.024	0.009	2.781	**0.012**
	2022	0.007	0.006	1.238	0.231

*Note:* *p* values for significant predictors are bolded.

Across all years, thicket cover was higher in fenced sites near the boundary. It occurred at similar proportions at fenced and unfenced sites farther from the boundary (Figure 4b). In 2018, 2019, and 2021, the thicket showed a significant positive relationship with distance from the boundary (*p* < 0.05). Forest/woodland cover showed a difference in proportion values of close to zero across all years and distances to boundary, indicating similar levels of forest cover in both sets of village lands (Figure 4b). Differences in proportion of cover of forest/woodland showed a significant but small positive relationship with distance to boundary in 2019, 2021, and 2022 (Table 1). Close to the boundary (0–1.4 km), forest cover in both IGR and SNP showed a slight increase after fencing (0.009 ± 0.007 km^2^ to 0.022 ± 0.003 km^2^ and 0.004 ± 0.001 km^2^ to 0.007 ± 0.002 km^2^, respectively). Thicket cover remained stable close to the boundary in both IGR and SNP (0.16 ± 0.03 km^2^ to 0.16 ± 0.02 km^2^ and 0.11 ± 0.01 km^2^ to 0.12 ± 0.02 km^2^).

### 3.2 Village Transects—Crop Types Variability

**FIGURE 5:**
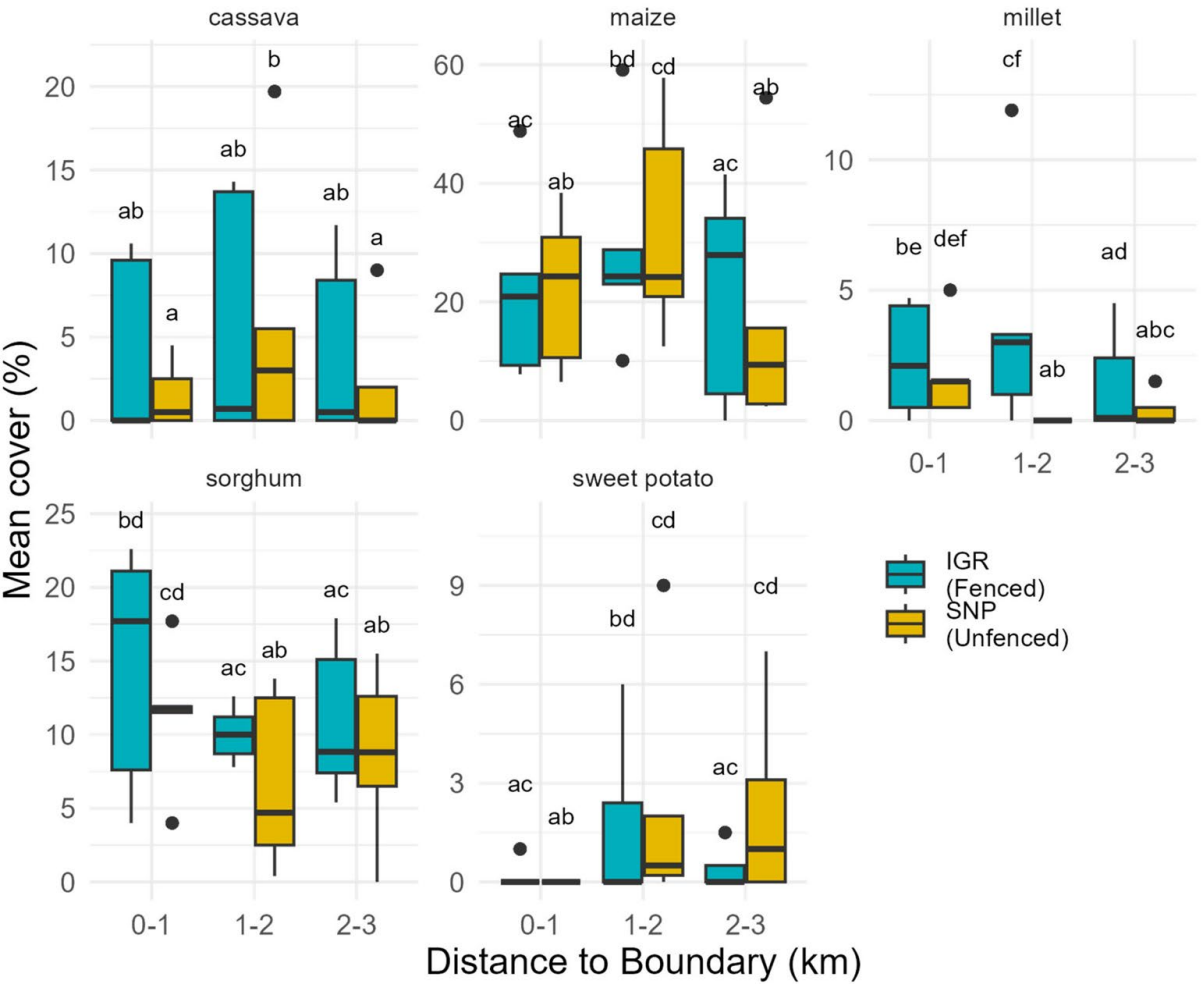
Percentage cover of area occupied by five key crops in 2022–2023 along three distance categories from the protected area boundary towards the village centers, between fenced and unfenced sites. Means with the same letter label are not significantly different, whereas means with different letter labels are. For instance, “a” vs. “ab” are considered different.

Model results showed higher AIC values and significant interactions between boundary type and distance to the boundary for all crops except sorghum (Appendices E and F). Accordingly, and to maintain consistency across analyses, the full interaction models were retained for all crop types. Our models revealed that the percentage coverage of cassava was unrelated to distance in fenced sites, whereas coverage in unfenced sites was higher at intermediate distances. In maize, a higher percentage of coverage was observed at intermediate distances in both fenced and unfenced areas. Millet showed a higher percentage near the boundary at the unfenced sites. Sorghum was planted with a higher percentage cover near the boundary for both fenced and unfenced sites. For sweet potatoes, in unfenced sites, there was a lower percentage coverage near the boundary than at intermediate and far distances. In contrast, in fenced sites, coverage was lower both near and far from the boundary, with higher values at intermediate distances (Figure 5).

### 3.3 Fencing and Crop Cultivation Relations

#### 3.3.1 Fence and Cropland Farming

FGD participants reported an increase in their involvement in crop cultivation in both dry and wet seasons of the year during the post‐fence stage compared to one‐season cultivation during the pre‐fence stage, attributing this change to enhanced security in their village lands, resulting in less crop damage by wildlife (especially elephants) and a reduced personal risk during crop farming due to encounters with elephants. Participants also shared that, before fencing, many youths were involved in wildlife poaching. However, during the post‐fence stage, they shifted their focus to crop cultivation and small businesses (Table 2). This suggests that the local community perceived the fence as having had significant positive impacts on both conservation and community livelihoods, as evidenced by changes in youth behavior, improved land security, and the promotion of sustainable agricultural practices.

**TABLE 2:** Sub‐themes, descriptions, and key quotes reveal the local community's attitudes towards electric conservation fencing in crop cultivation.

Sub‐themes	Descriptions	Illustrative quotes
Crop cultivation	Address issues related to changing youth behavior regarding illegal wildlife hunting.	“In the past, many youths were illegal wildlife hunters, but nowadays, they are primarily engaged in crop farming and small businesses. Many people were jailed [for poaching], which negatively impacted their families' livelihoods.” “Before the fence, we only cultivated one season per year, but after the fence was constructed, we now cultivate twice a year, in which youths spend more time in crop farming than poaching.”
	Addressing issues of village land security	“During the pre‐fence stage, much of our time was spent guarding crop fields, but now we have more time for cultivation.” “We are glad that elephants are no longer a problem, but it would be even better if the fence could be improved also to keep out other problem animals such as baboons, monkeys, and hyenas from village land, as they are still posing problems to us.”
	Promoting food security	“Some local farmers have even expanded their farming areas from 1–3 to 5–10 ha.” “My husband was killed inside this reserve due to poaching, and I struggled to raise the children myself due to lack of enough food to feed them, but after the construction of this fence, it was the first time I managed to secure enough food for my family, so I treat this fence as my second God.”
Crop field demarcation	Addressing crop field demarcation conflicts and diversifying local income	“Before the fence, sisal was rarely used for marking crop field boundaries as it was like a soup to elephants, but nowadays, sisals are widely used for demarcating crop fields. This helps us to avoid conflicts with neighbors or with livestock keepers.” “Apart from crop field demarcation, the use of sisal fences serves as an income source, as there are buyers who purchase them for use in their factories.”
Elephant‐preferred crops	Addressing the cultivation of crops preferred by elephants near the protected area boundary	“Nowadays, people grow cassava near the protected area, a crop that takes a long time to mature and was previously vulnerable to elephant raids, requiring significant defense efforts. We have also seen an increase in the cultivation of horticultural crops like carrots, tomatoes, and eggplants, which was impossible in the past.”

#### 3.3.2 Fence and Crop Field Demarcation

Local communities demarcate their crop fields to prevent livestock from entering and to avoid conflicts with neighbors. Sisal plantations are natural systems that serve as effective boundary markers, providing income for investors who purchase them as raw materials. However, despite these benefits, crop farmers avoided using sisal for field demarcation because they believed elephants are attracted to it and that it is less effective than tree branches. In the post‐fence stage, however, sisal has become a widely used material for demarcating field boundaries (Table 2).

#### 3.3.3 Fence and Elephant‐Preferred Crops

Participants reported that during the post‐fence stage, local communities expanded their crop fields and began cultivating high‐value crops closer to the fence line, a practice that had not been feasible during the pre‐fence stage (Table 2).

### 3.4 Fencing and Land Use Conflict Relations

#### 3.4.1 Resettlement and Land Use Accessibility

While the fence has had a positive impact on crop farmers, few livestock keepers with large herds of cattle have voluntarily moved to unfenced villages because the fence prevents them from taking livestock into the PA for illegal grazing. In addition, participants reported that the fence restricts their access to grasses within the reserve, which they traditionally used to thatch (roof) their houses, despite such activities being illegal. As an alternative, participants mentioned that they often seek permission from the nearby Tabora B Prison Management to access their rangeland, where they are allowed to harvest thatching grass free of charge (Table 3).

**TABLE 3:** Sub‐themes, descriptions, and key quotes illustrate the relationships among electric conservation fencing, human resettlement, access to resources within the protected area, and potential land‐use conflicts.

Sub‐themes	Descriptions	Illustrative quotes
Land use accessibility	The unintended, voluntary resettlement of livestock keepers with large herds occurred after the fence restricted their access to protected areas, leading to illegal grazing. Most local communities are positively affected by the fence and engage in subsistence farming, including keeping a small number of livestock. Addressing illegal livestock grazing and access to resources found inside the protected area	“Livestock keepers with large herds dislike the fence because they previously grazed their livestock illegally inside the protected area. Although they are few (less than 1% of the population) and not indigenous to these villages, many have moved to villages like Issenye, Nata, Guikongo, and Tamkeri, where there is no fence between the village land and the protected area.” “More than 99% of the local people here depend on subsistence farming, cultivating crops, and raising a small number of livestock. My village lies at the end of this fence, and many people are still suffering from elephant attacks. The owners of the large herds of livestock do not live here; they only show up occasionally during meetings to oppose the fence, then leave their livestock in the care of herders.” “Sometimes it is challenging to cross the fence to access thatching grass and water inside the reserve. However, people are permitted to harvest thatching grass in the Tabora B Prison Rangeland free of charge, provided they submit a request to the Prison Management. If caught doing so without permission, one will be fined ten thousand Tanzania shillings.”
Land use conflicts	Livestock keepers expressed dissatisfaction with the prohibitions on accessing protected areas, which they had previously used for illegal grazing. They also raised concerns about expanding croplands into communal grazing areas, which violates established land‐use plans and further limits their access to grazing resources.	“As the chairperson of this village, I have recently been constantly mediating conflicts with people living near the fence line.” “After fencing, crop farmers have started cultivating areas designated for communal livestock grazing, which creates another problem. Livestock keepers are complaining about this action, as it contradicts our land use plans.”

#### 3.4.2 Land Use Conflicts

Participants reported that the fence contributed to the expansion of croplands into communal livestock grazing areas, restricted access to grazing resources for livestock keepers, escalated conflicts with crop farmers, and violated village land‐use plans (Table 3).

## 4 Discussion and Conclusion

The deployment of electric conservation fencing is an Africa‐wide management intervention to mitigate human–wildlife conflicts and protect biodiversity (especially endangered species). Though the effects of installing an electric fence in the context of wildlife and human–wildlife conflict are well‐studied, the impacts of electric fencing on land‐use changes in community lands bordering PAs are less well known. In this study, we find that electric conservation fencing strongly affected land‐use patterns on village lands bordering the fenced PA and had strong spillover effects in neighboring village lands where no fence was installed.

We observed a stabilized relative proportion of cropland and grassland within 1.4 km from the fence line in village lands bordering the fence, and an increase in grassland cover within 1.4 km from the unfenced PA boundary at the expense of cropland. This observation aligns with communities reporting increased crop security on village lands bordering the fence line, leading to increased cultivation in both seasons near the protected area boundary and an increase in horticultural crops such as tomatoes, carrots, and eggplants. By reducing wildlife interference and improving land security, particularly after the exclusion of elephants from human settlements, the fence has enabled safer, more diverse agricultural practices near protected areas, supporting the majority of the local population. This finding aligns with research from other landscapes, where fencing has been shown to reduce wildlife‐caused crop damage and increase crop yields (Graham, Douglas‐Hamilton, et al. 2009; Graham, Notter, et al. 2009; Davies et al. 2011). Communities reported that some livestock keepers with large herds moved to unfenced villages after the fence was introduced, as they could no longer graze illegally inside the reserve because the fence prohibited it. This shift may explain the increased grassland cover in villages bordering the nearby unfenced protected area, which aligns with the lower proportion of area burned near the unfenced protected area boundary (Appendix G). While this suggests that fencing is successful in reducing illegal livestock incursions into protected areas (a key conservation challenge in the region, Denninger Snyder et al. 2019), it also shows that the problem has been moved and increases pressure on nearby unfenced protected areas, a situation that has been observed in other studies (Oliveira et al. 2007; Ewers and Rodrigues 2008).

Surprisingly, although we found that fencing stabilized agricultural cover near the boundary compared to unfenced sites, we did not observe any apparent effects of fencing on the cultivation of elephant‐preferred crops. Our study found that different crops exhibit distinct spatial patterns of boundary proximity, with several showing peak coverage at intermediate distances (e.g., cassava, maize, and sweet potato in fenced sites), while others, such as millet and sorghum, tend to be concentrated closer to boundaries. Cassava, maize, and sorghum are key staple crops cultivated in sub‐Saharan Africa, including our study area, serving diverse populations as part of their dietary intake (Rurinda et al. 2014) and as a source of income (Kangile et al. 2020). Previous studies have documented how fencing can create a sense of security, allowing crop farmers to cultivate their preferred crops (Thouless and Sakwa 1995; O'Connell‐Rodwell et al. 2000). However, the Ikorongo fence was designed to reduce elephant‐related crop damage by 79%, not the 19% attributed to vervets and baboons (Denninger Snyder et al. 2023). As these animals can still pass through the fence line, this could have discouraged farmers from cultivating several crops (Alemayehu and Tekalign 2020), and the choice of crop type may be influenced by factors other than wildlife damage, including proximity to the PA boundary.

Participants also described an encouraging outcome of the fence's presence: improved youth behavior and a focus on cultivation and small businesses, which could have contributed to stabilized crop cultivation by devoting time to business activities. Before the introduction of the fence, the area close to the northern boundary of IGR was known for its high poaching rates (Mfunda and Røskaft 2010; Denninger Snyder et al. 2019). Although we did not specifically measure post‐fencing poaching rates, community interviews described a shift among youths from wildlife hunting to crop cultivation and small businesses, highlighting the fence's potential to align community livelihoods with conservation goals. Not only can this behavioral change reduce poaching pressures, but it can also enhance livelihoods among local communities, which is critical for the long‐term success of conservation initiatives (Roe et al. 2015).

Community members also noted new, though less intense (i.e., minor conflict), land‐use conflicts following the introduction of the conservation fence. Crop farmers began encroaching on communal grazing areas, highlighting the challenge of balancing competing land‐use needs. This observation underscores the importance of inclusive land‐use management that considers the needs of both crop farmers and livestock keepers when implementing electric conservation fences or other measures to enhance security on village lands against wildlife, which frequently cause crop/livestock losses (Western et al. 2009). Furthermore, the fence prevented local communities living adjacent to the fence line from accessing thatching grass and water within the reserve, even though it was illegal. Other studies found that local communities risk their lives to access resources in the protected areas mainly due to poverty (Evans and Adams 2016; Foya et al. 2023; Zisadza et al. 2025), which highlights the importance of addressing basic needs and encouraging quality housing (e.g., iron sheets) for communities living near protected areas to reduce the need for so‐called “illegal incursions”.

Overall, this study's findings highlight that the impacts of conservation fencing are complex and have significant implications for conservation policies and practices. While fencing can enhance village land security from crop‐ and livestock‐damaging animals like elephants and support sustainable agriculture for many local people, it may also lead to unintended consequences, such as increased livestock encroachment into adjacent areas and novel land‐use conflicts for a few livestock keepers with large herds. The differential impacts of this 30 km electric conservation fence on subsistence farmers and a few livestock keepers underscore both the importance and the challenge of adopting a holistic approach that considers the needs of all stakeholders while also reducing illegal activities inside protected areas. In conclusion, conservation fencing could be complemented by improved land‐use management strategies, enforcement of existing land‐use plans, promotion of low‐density livestock keeping, and support for community livelihood, especially for households unintentionally affected by the fence.

This study was subject to several limitations. Firstly, distinguishing between certain land‐cover classes—such as heavily grazed grassland versus sparsely cultivated fields—was challenging due to spectral similarities and the coarse‐to‐moderate resolution of the imagery, even when supplemented with ground‐truthing transects. Secondly, community‐reported land‐use changes may reflect perception‐based biases influenced by socio‐economic or political factors, which could affect the interpretation of drivers behind observed LULC patterns. Thirdly, the study's quasi‐experimental design—comparing fenced and unfenced sites before and after fence construction—cannot fully isolate the impacts of fencing from broader landscape processes such as market‐driven land expansion, policy shifts, or demographic change. Finally, given the increasing human populations and activities near protected areas across sub‐Saharan Africa, including Tanzania, and the relatively short study period (2019–2022) we used, it is likely that land‐use and land‐cover changes will become more pronounced over time. Therefore, reassessing the impacts of this fence over a longer time frame is warranted in future studies. Despite these limitations, integrating remote sensing, field surveys, and local knowledge provides a robust foundation for understanding landscape changes around the fencing intervention.

## Author Contributions

**Michael Honorati Kimaro:** writing – original draft (equal). **Milenka Ishasha Sloots:** writing – original draft (equal). **Kristen Denninger Snyder:** writing – original draft (equal). **Noel Latiaeli Mbise:** writing – original draft (equal). **Victor Alexander Kakengi:** writing – original draft (equal). **Walter Di Nicola:** writing – original draft (equal). **Han Olff:** writing – original draft (equal).

## Funding

This project was funded by the Grumeti Fund, Tanzania, and the University of Groningen via the Ubbo Emmius Fund, Netherlands (grant no. 201383/190240126).

## Ethics Statement

This study was conducted in accordance with the ethical guidelines outlined in the COSTECH permits (2022‐388‐NA‐2015‐124 and 2023–802‐ER‐2015‐124) issued for the research.

## Conflicts of Interest

The authors declare no conflicts of interest.

## Data Availability

All the required data and R scripts are provided via https://drive.google.com/drive/folders/1RdHQ‐Af4nX_kPLQW8ZgiXaXzgm_sa5AS?usp=sharing.

## Appendix A Satellite Imagery Sources

**Table:**

Year	Date range February	Date range wet season	Date range dry season	Sentinel 2 product used	Tiles used	Sentinel 1 product used
2018	31 Jan to 01 March	30 April to 01 June	30 July to 01 Sep	Sentinel 2, Level 1C	36MXD, 36MXC	Sentinel 1A, DV Polarization, Ascending Orbit
2019	31 Jan to 01 March	30 April to 01 June	30 July to 01 Sep	Sentinel 2, Level 2A	36MXD, 36MXC	Sentinel 1A, DV Polarization, Ascending orbit
2021	31 Jan to 01 March	30 April to 01 June	30 July to 01 Sep	Sentinel 2, Level 2A	36MXD, 36MXC	Sentinel 1A, DV Polarization, Ascending orbit
2022	31 Jan to 01 March	30 April to 01 June	30 July to 01 Sep	Sentinel 2, Level 2A	36MXD, 36MXC	Sentinel 1A, DV Polarization, Ascending orbit

## Appendix C LULC Types Identified in Village Land Transects, Including the Broader Category and Each LULC Type Fits

**Table:**

LULC type	LULC category	Explanation (if necessary)
House	Built‐up	
Bare	Built‐up	Bare ground was classified as built‐up because it was observed only around houses and on roads.
Kopje	Kopje	
Water	Water	
Fallow	Fallow	The land was considered fallow if there were indications it had previously been used as cropland but was now overgrown.
Grass	Grass	Grass was considered separate from vegetation as it was, in many cases, managed as grazing land, whereas vegetation was not necessarily associated with a specific land use.
Reed	Vegetation	
Tree	Vegetation	
Shrub	Vegetation	Any tree less than 3 m in height was considered a shrub
Sorghum	Food crops	Crops used by the grower for food/subsistence farming
Maize	Food crops	Crops used by the grower for food/subsistence farming
Sunflower	Food crops	Crops used by the grower for food/subsistence farming
Millet	Food crops	Crops used by the grower for food/subsistence farming
Collard Greens	Food crops	Crops used by the grower for food/subsistence farming
Sugarcane	Food crops	Crops used by the grower for food/subsistence farming
Cassava	Food crops	Crops used by the grower for food/subsistence farming
Beans	Food crops	Crops used by the grower for food/subsistence farming
Sweet Potato	Food crops	Crops used by the grower for food/subsistence farming
Groundnut	Cash crops	Cash crops are those produced for commercial value rather than for subsistence farming.
Sesame	Cash crops	Cash crops are those produced for commercial value rather than for subsistence farming.
Plowed	Plowed	Any plowed field in which crops were not (yet) visible was considered plowed.
Banana	Horticultural crops	Horticultural crops include fruits, vegetables, medicinal, ornamental, and aromatic plants.
Papaya	Horticultural crops	Horticultural crops include fruits, vegetables, medicinal, ornamental, and aromatic plants.
Mango	Horticultural crops	Horticultural crops encompass a diverse range of plants, including fruits, vegetables, medicinal, ornamental, and aromatic plants.
Avocado	Horticultural crops	Horticultural crops encompass a diverse range of plants, including fruits, vegetables, medicinal, ornamental, and aromatic plants.
Orange	Horticultural crops	Horticultural crops encompass a diverse range of plants, including fruits, vegetables, medicinal, ornamental, and aromatic plants.
Tomato	Horticultural crops	Horticultural crops encompass a diverse range of plants, including fruits, vegetables, medicinal, ornamental, and aromatic plants.
Onion	Horticultural crops	Horticultural crops encompass a diverse range of plants, including fruits, vegetables, medicinal, ornamental, and aromatic plants.

## Appendix D

Checklist of themes discussed during the focus group discussions and key informant interviews with local village leaders bordering the fence line, district politicians of the ruling party, and district council officials who engage in community development and environmental issues.

1. State why we want to present research findings and get their insights about the fence (For Focus Group Discussion and Key Informants)
2. Seek Consent from participants about video recording and state other ethical considerations (For Focus Group Discussion and Key Informants)
3. Overview of HWC in Tanzania and why this fence is important (for Focus Group Discussion and Key Informants)
  - General project observation
  - Time of research and dissemination period
  - The difference or uniqueness of the Ikorongo Fence compared to other fences in other areas
  - Outcome of the electric fence on HWC
  - What should be done as per the existing National HWC Mitigation Strategy 2020–2024
4. Share research findings (for Focus Group Discussion and Key Informants)
  - Community perceptions obtained from the systematic interviews
  - Response to wildebeest migration along the fence
  - Impacts of the fence on wildlife structure and diversity
  - Impacts of the fence on land use and land cover based on remote sensing analyses
  - Impacts of the fence on vegetation structure and diversity
  - Impacts of the fence on wildlife mortalities and fence vandalism
  - Other researchers assessed the effects of this fence, and their findings
5. Grumeti Fund Management (for Focus Group Discussion and Key Informants)
  - Impacts of the fence on elephant movements
  - Fence construction considerations and the cost incurred
  - Fence maintenance costs and challenges
  - Plans
6. Social part based on the villagers (for Focus Group Discussion)
  - Benefits and costs of this fence—testimonials from village leaders, women, and youth living adjacent to this fence
  - Witnesses of HWC incidents before and after fencing
  - Political testimonials about this fence
  - Vandalism and protection of the fence
7. Government support (Serengeti district office; for Key Informant Interviews)
  - Impacts of the fence on community properties and security
  - Experience with HWC before and after fencing
  - The efforts the government devotes to addressing HWC in this district, and what changes the fence is contributing to
  - How does the community and district management perceive this fence
  - Any new challenges that emerged after the installation of the fence
  - What are the existing plans for addressing HWC in this district
8. Political support (Ruling party politicians; for Key Informant Interviews)
  - What is the current HWC situation in this district, and how does it affect the relationship with local people and the government
  - What efforts have been devoted to addressing HWC in this district
  - Which efforts have been addressed, and which are still pending
  - What are the positives and negatives of this fence
  - Who constructed this fence, and who initiated the discussion of fencing
  - What are the newly emerged challenges after the installation of this fence
  - What are the existing plans for addressing HWC in this district

## Appendix E

Table showing model AIC rankings for full interaction between distance to boundary and boundary type, and no interaction between distance to boundary and boundary type. Rows in bold indicate the best model.

**Table:**

Crop type	Model type	AIC
Maize	No interaction between distance to boundary and boundary type	2271.899
**Maize**	**Full interaction between distance to boundary and boundary type**	**2267.702**
Millet	No interaction between distance to boundary and boundary type	1221.498
**Millet**	**Full interaction between distance to boundary and boundary type**	**1169.382**
**Sorghum**	**No interaction between distance to boundary and boundary type**	**1658.003**
Sorghum	Full interaction between distance to boundary and boundary type	1660.509
Cassava	No interaction between distance to boundary and boundary type	1636.789
**Cassava**	**Full interaction between distance to boundary and boundary type**	**1634.380**
Sweet potato	No interaction between distance to boundary and boundary type	1231.222
**Sweet potato**	**Full interaction between distance to boundary and boundary type**	**1222.073**

## Appendix F

Table showing the coefficients of the fixed effects of full interaction models (meancover ~ distance to boundary * boundary type + (1|transect)). Significant terms are marked in bold.

**Table:**

Term	Estimate	Standard error	df	*t*	Pr(>|*t*|)
*Cassava*					
Intercept	4.040	2.019	8.684	2.001	0.078
Boundary type (unfenced)	−2.540	2.856	8.684	−0.890	0.398
Distance to boundary (1‐2 km)	1.700	0.705	281.998	2.410	**0.017***
Distance to boundary (1‐2 km) × Boundary type (unfenced)	2.440	0.998	281.998	2.446	**0.015***
Distance to boundary (2‐3 km)	0.039	0.723	282.071	0.054	0.957
Distance to boundary (2‐3 km) × Boundary type (unfenced)	0.661	1.010	282.035	0.654	0.513
*Maize*					
Intercept	22.300	6.522	8.533	3.419	**0.008****
Boundary type (unfenced)	−0.160	9.223	8.533	−0.017	0.987
Distance to boundary (1‐2 km)	6.760	2.049	281.986	3.300	**0.001****
Distance to boundary (1‐2 km) × Boundary type (unfenced)	3.340	2.897	281.986	1.153	0.250
Distance to boundary (2‐3 km)	−0.195	2.098	282.045	−0.093	0.926
Distance to boundary (2‐3 km) × Boundary type (unfenced)	−5.025	2.933	282.016	−1.714	0.088
*Millet*					
Intercept	2.340	0.764	9.035	3.062	**0.013***
Boundary type (unfenced)	−0.540	1.081	9.035	−0.500	0.629
Distance to boundary (1‐2 km)	1.500	0.324	281.995	4.632	**0.000*****
Distance to boundary (1‐2 km) × Boundary type (unfenced)	−3.300	0.458	281.995	−7.206	**0.000*****
Distance to boundary (2‐3 km)	−0.976	0.332	282.104	−2.943	**0.004****
Distance to boundary (2‐3 km) × Boundary type (unfenced)	−0.424	0.464	282.051	−0.914	0.361
*Sorghum*					
Intercept	14.600	1.885	8.883	7.745	**0.000*****
Boundary type (unfenced)	−4.540	0.740	282.000	−6.132	**0.000*****
Distance to boundary (1‐2 km)	−3.791	0.758	282.094	−4.999	**0.000*****
Distance to boundary (1‐2 km) × Boundary type (unfenced)	−3.220	2.666	8.883	−1.208	0.258
Distance to boundary (2‐3 km)	−0.060	1.047	282.000	−0.057	0.954
Distance to boundary (2‐3 km) × Boundary type (unfenced)	1.091	1.060	282.048	1.029	0.304
*Sweet Potato*					
Intercept	0.200	0.562	10.672	0.356	0.729
Boundary type (unfenced)	−0.200	0.794	10.672	−0.252	0.806
Distance to boundary (1‐2 km)	1.480	0.359	281.981	4.117	**0.000*****
Distance to boundary (1‐2 km) × Boundary type (unfenced)	0.860	0.508	281.981	1.692	0.092
Distance to boundary (2‐3 km)	0.350	0.368	282.249	0.951	0.342
Distance to boundary (2‐3 km) × Boundary type (unfenced)	1.870	0.515	282.119	3.634	**0.000*****

## Appendix G

The graph illustrates the area burned and rainfall (calculated from January to May) for the period from 2018 to 2022 at various distances from the northern boundaries of the Ikorongo Game Reserve (fenced) and Serengeti National Park (unfenced). The vertical dotted lines represent the completion of the fence in April 2020. The lines before April 2020 refer to the pre‐fence stage, while those after April 2020 refer to the post‐fence stage. The distance from the PA boundaries was divided into four intervals, spanning up to 10 km for the pre‐fence and post‐fence stages: 0–0.5, 0.5–1, 1–5, and 5–10 km. Using Google Earth Engine, we quantified the total area burned in the study area using the MODIS/Terra+ Burned Area Monthly remote sensing product, retrieved from https://developers.google.com/earth‐engine/datasets/catalog/MODIS_061_MCD64A1. Rainfall was calculated from the UCSB‐CHG_CHIRPS_PENTAD dataset, retrieved from https://developers.google.com/earth‐engine/datasets/catalog/UCSB‐CHG_CHIRPS_PENTAD.
